# Granulocyte-macrophage colony-stimulating factor alone or with dacarbazine in metastatic melanoma: a randomized phase II trial

**DOI:** 10.1054/bjoc.2001.2120

**Published:** 2001-11

**Authors:** A Ravaud, M Delaunay, C Chevreau, V Coulon, M Debled, C Bret-Dibat, F Courbon, N Gualde, B Nguyen Bui

**Affiliations:** Department of Medicine, Institut Bergonié, Regional Cancer Center, Bordeaux, France; UMR 5540 CNRS, Bordeaux, France; Department of Dermato-Oncology, Hôpital Saint-André, Bordeaux, France; Department of Medicine, Institut Claudius Regaud, Regional Cancer Center, Toulouse, France; Laboratory of Immunology, Institut Bergonié, Regional Cancer Center, Bordeaux, France

**Keywords:** melanoma, GM-CSF, DTIC, clinical trial

## Abstract

The potential antitumoural effect of granulocyte-macrophage colony-stimulating factor (GM-CSF) led us to evaluate GM-CSF alone or with dacarbazine (DTIC) in metastatic melanoma in first line randomized phase II. Treatment was arm A: GM-CSF: 5 μg kg^−1^, bid, 14 consecutive days every 21 days and arm B: GM-CSF: 5 μg kg^−1^, bid, day 2 to day 19 every 21 days and DTIC: 800 mg m^−2^, day 1 of each cycle. 32 patients (pts) were included, 15 pts in arm A and 17 in arm B. All pts had visceral metastatic sites. 9 had only one metastatic site. The median number of cycles given was 2 in arm A and 3 in arm B. 100% and 89.4% of the planned dose of GM-CSF was given in arm A and arm B respectively. No objective response was obtained. 19 pts experienced at least WHO grade 3 toxicity. All pts had fever, 29 had a decrease in performance status and 23 had pain. Grade 3 toxicity were fever (38.7%), decrease in performance status (32.3%), pain (19.4%) and dyspnoea (12.5%). In this study, GM-CSF alone or in association with DTIC did not induce any antitumoural activity with subsequent toxicity. © 2001 Cancer Research Campaign   http://www.bjcancer.com


*British Journal of Cancer* (2001) **85**(10), 1467–1471
© 2001 Cancer Research Campaign
doi: 10.1054/ bjoc.2001.2120, available online at http://www.idealibrary.com on IDEAL

### Granulocyte-macrophage colony-stimulating factor alone or with dacarbazine in metastatic melanoma: a randomized phase II trial

**A Ravaud[1,2], M Delaunay[3], C Chevreau[4], V Coulon[2,5], M Debled[1], C Bret-Dibat[3], F Courbon[4], N Gualde[2,5] and B Nguyen Bui[1]**

[1]Department of Medicine, Institut Bergonié, Regional Cancer Center, Bordeaux, France; [2]UMR 5540 CNRS, Bordeaux, France; [3]Department of Dermato-Oncology, Hôpital Saint-André, Bordeaux, France; [4]Department of Medicine, Institut Claudius Regaud, Regional Cancer Center, Toulouse, France; [5]Laboratory of Immunology, Institut Bergonié, Regional Cancer Center, Bordeaux, France

**Summary** The potential antitumoural effect of granulocyte-macrophage colony-stimulating factor (GM-CSF) led us to evaluate GM-CSF alone or with dacarbazine (DTIC) in metastatic melanoma in first line randomized phase II. Treatment was arm A: GM-CSF: 5 µg kg$^{-1}$, bid, 14 consecutive days every 21 days and arm B: GM-CSF: 5 µg kg$^{-1}$, bid, day 2 to day 19 every 21 days and DTIC: 800 mg m$^{-2}$, day 1 of each cycle. 32 patients (pts) were included, 15 pts in arm A and 17 in arm B. All pts had visceral metastatic sites. 9 had only one metastatic site. The median number of cycles given was 2 in arm A and 3 in arm B. 100% and 89.4% of the planned dose of GM-CSF was given in arm A and arm B respectively. No objective response was obtained. 19 pts experienced at least WHO grade 3 toxicity. All pts had fever, 29 had a decrease in performance status and 23 had pain. Grade 3 toxicity were fever (38.7%), decrease in performance status (32.3%), pain (19.4%) and dyspnoea (12.5%). In this study, GM-CSF alone or in association with DTIC did not induce any antitumoural activity with subsequent toxicity. © 2001 Cancer Research Campaign http://www.bjcancer.com

**Keywords**: melanoma; GM-CSF; DTIC; clinical trial

Granulocyte-macrophage colony-stimulating factor (GM-CSF) is a haematopoietic growth factor acting on early multipotent haematopoietic progenitors. GM-CSF regulates growth and differentiation to macrophages or neutrophils (Metcalf, 1985; Sieff, 1987). In prospective randomized trials, GM-CSF has shown a significant increase in neutrophil count and a significant reduction of neutropenia duration after chemotherapy or autologous bone marrow transplantation (Greenberg et al, 1996; Update of recommendations for the use of haematopoietic colony-stimulating factors: evidence-based clinical practice guidelines, 1996; Ravaud et al, 1998). Moreover, GM-CSF induces mobilization and facilitates collection of peripheral blood stem cells (Demirer et al, 1996).

Furthermore, there is some evidence that GM-CSF could have antitumoural activity by enhancing immune response. In vitro GM-CSF activates monocytes and macrophages to be cytotoxic to human cell lines (Grabstein et al, 1986; Mazumder et al, 1990; Williams et al, 1994). GM-CSF induces antibody-dependent cell-mediated cytotoxicity (Kushner and Cheung, 1989; Mazumder et al, 1990; Nagler et al, 1994). Proliferation of interleukin-2 driven T lymphocytes is enhanced in the presence of GM-CSF (Santoli et al, 1988).

In experimental animal models, GM-CSF has been found to enhance the cytotoxicity of macrophages through production of nitric oxide and free radicals (Hill et al, 1993). Moreover, gene transfection of GM-CSF into tumour cells induces an antitumour activity and protection of mice when challenged with tumours (Dranoff et al, 1993). In humans, GM-CSF enhances cytotoxicity of T lymphocytes CD3+ CD16- following bone marrow transplantation (Richard et al, 1993). Antibody-dependent cell-mediated cytotoxicity is also increased (Ragnhammar et al, 1993). The ability to stimulate presentation of antigens is supported by GM-CSF enhancing MHC class II expression on monocytes (Willman et al, 1989). Moreover, GM-CSF is one of the main cytokines used for producing ex vivo dendritic cells, which act as professional antigen-presenting cells to induce T cell immune response (Szabolcs et al, 1995; Bender et al, 1996; Coulon et al, 2000). When GM-CSF was used during antitumoural vaccination with peptide in patients with melanoma, it enhanced the immune response through activation of CD4 + and CD8 + T cells (Jager et al, 1996).

The efficacy of GM-CSF as an antitumour agent has been hypothesized in a prospective trial in patients with soft-tissue sarcomas treated with chemotherapy in association with GM-CSF for prevention of haematological toxicity. One study showed the unexpected frequency of partial and complete response that could account for an antitumoural effect of molgrastim (Edmonson et al, 1994, 1997).

Prognosis of metastatic melanoma is poor and median survival is 6–8 months (Balch et al, 1997). Palliative treatment is based on chemotherapy with dacarbazine as first-line treatment (Ho, 1995). The arguments suggesting the efficacy of non-specific immunotherapy in melanoma are that interleukin 2 has been shown to induce clinical responses and long durable responses in

*Received 7 February 2001*
*Revised 26 July 2001*
*Accepted 30 July 2001*

*Correspondence to:* A Ravaud metastatic melanoma (Legha et al, 1998; Atkins et al, 1999) and that interferon alpha induces a significant increase of disease-free survival and a possible increase in survival when used as adjuvant treatment (Kirkwood et al, 1996).

Due to a possible antitumoural effect of GM-CSF through an immune modulation and to low efficacy of chemotherapy in metastatic melanoma, we conducted a multicenter randomized trial comparing GM-CSF alone and GM-CSF associated with dacarbazine in metastatic melanoma patients.

### PATIENTS AND METHODS

### Patients

Adult patients under 70 years of age were eligible if they had histologically confirmed melanoma with distant metastatic disease that could be measured in 2 dimensions. Patients had an Eastern Cooperative Oncology Group (ECOG) performance status ≤ 2. No prior chemotherapy was allowed. Previous adjuvant interferon alpha was allowed, but a delay of more than 6 months was required before inclusion. Patients with central nervous system metastases were excluded, except if they had been previously treated (surgery and/or radiotherapy) and subsequently demonstrated stable disease or no evidence of recurrence. Biological inclusion criteria were leukocyte count $\geq 4 \times 10^9$ $l^{-1}$, haematocrit level ≥ 30%, platelet count $\geq 100 \times 10^9$ $l^{-1}$, creatinine concentration < 150 μmol $l^{-1}$ and transaminases < 2-fold the upper limit of normal values except in the case of hepatic metastases where < 5 fold the upper limit of normal values was required. Furthermore, patients with severe infection or positivity for HIV or hepatitis B or C were not eligible. Glucocorticosteroids were not allowed. Patients did not have any additional malignancy other than cervical carcinoma in situ or basal cell carcinoma. Pregnant or lactating women were excluded. Patients had to give written informed consent before entry into the trial.

### Pretreatment evaluation

Clinical history and physical examination were recorded for all patients. Before inclusion, patients had a staging including thoracic and abdominal computed tomographic scans and a chest X-ray.

### Study design and statistical considerations

The study was an unblinded randomized phase II trial conducted in 3 centres with no stratification. Registration and randomization of eligible patients were performed at the data monitoring centre of Institut Bergonié. The primary end point was the objective response rate, and secondary end points were response duration, toxicity and the response with dacarbazine alone after failure with GM-CSF alone.

An objective response rate of at least 20% would be of sufficient interest to encourage further investigation of GM-CSF. The determination of the numbers of patients followed Gehan's rules, which indicates that including 14 patients gives a 95% chance of detecting at least one response if the response rate was ≥ 20% (Gehan, 1961). If at least one response occurred in the first 14 patients, it was planned to increase the number of patients up to a total of 25 patients per arm to assess the response rate more precisely.

### Treatment

Eligible patients were randomly assigned to receive either GM-CSF or GM-CSF with decarbazine (GM-CSF + DTIC). Dosage and schedule of GM-CSF were based on the trial of Edmonson et al (1994, 1997). Patients in the GM-CSF arm received subcutaneous GM-CSF at a dose of 5 μg $kg^{-1}$, bid for 14 consecutive days every 21 days. Patients in the GM-CSF + DTIC arm received DTIC at a dose of 800 mg $m^{-2}$ on day one and subcutaneous GM-CSF at a dose of 5 μg $kg^{-1}$, bid from day 2 to day 19 every 21 days.

Response was assessed every 2 cycles. Work-up was identical to the initial pretreatment evaluation. Each time stabilization or an objective response occurred, 2 additional cycles of treatment were planned up to a total of 12 cycles.

Patients who progressed under GM-CSF alone received DTIC at a dose of 800 mg $m^{-2}$ on day one every 21 days.

### Evaluation of treatment

The World Health Organization (WHO) criteria were used to determine tumour response (Miller et al, 1981). Complete response (CR) was defined as the complete disappearance of all measurable and evaluable tumour sites for at least 4 weeks. The duration of CR was calculated from the first date of documentation of CR to the date of the first evaluation of disease progression. Partial response (PR) was considered to be a ≥ 50% decrease in the sum of products of the greatest perpendicular diameters that lasted for at least 4 weeks, with no increase in known lesions and without appearance of any new lesions. The duration of PR was calculated from the first day of treatment. When the evaluation showed a decrease in lesions < 50% and an increase < 25%, patients were considered to have a stable disease (SD). Progressive disease (PD) was considered to be when any lesion increased by ≥ 25% or when a new lesion appeared. Patients who presented with a CR, PR or SD were further evaluated every 2 to 3 months during the first year and then every 4 to 6 months. Survival duration was evaluated from the start of treatment to the date of the last contact or the date of death. Toxicities encountered were classified according to the World Health Organization grading system.

### RESULTS

### Patient characteristics

32 patients were entered into this study (Table 1). All were evaluable for toxicity. 2 were considered not to be assessable for tumour response: one had a grade 3 decrease in general status and went off the study, and another refused to continue due to grade 3 pain after one cycle of treatment.

### Administration of treatment and toxicity

During treatment, the median dose of GM-CSF given in the GM-CSF arm was 8960 μg (range: 3300–11 200) corresponding to 100% of the planned dose, while the median dose of GM-CSF in the GM-CSF + DTIC arm was 12 070 μg (range: 7820–17 340), accounting for 89.4% of the planned dose. The median number of cycles received was 2 in the GM-CSF arm and 3 in the GM-CSF + DTIC arm. 3 patients (20%) in the GM-CSF arm and 11 (64.7%) in the GM-CSF + DTIC arm had a dose modification of GM-CSF due to the induced toxicity. 6 patients (40%) in the GM-CSF arm

 *© 2001 Cancer Research Campaign*

**Table 1** Patient characteristics

| Characteristics | GM-CSF arm | GM-CSF + DTIC arm |
|---|---|---|
| No. of patients | 15 | 17 |
| Sex (male/female) | 11/4 | 11/6 |
| Age, y. Median (range) | 56 (35.5/70.5) | 57 (36.6/67.6) |
| Site of melanoma | | |
| Cutaneous | 11 | 12 |
| Ocular | 1 | 2 |
| Visceral | 1 | 0 |
| Unknown | 2 | 3 |
| Time from diagnosis to metastasis Median (range) months | 17.5 (0/89) | 17.9 (0/140.5) |
| ECOG performance status | | |
| 0 | 7 | 7 |
| 1 | 7 | 8 |
| 2 | 1 | 2 |
| Pts with visceral metastatic sites | 15 | 17 |
| Sites of metastatic disease | | |
| Lung | 7 | 13 |
| Superficial lymph nodes | 1 | 2 |
| Deep lymph nodes | 6 | 9 |
| Liver | 5 | 7 |
| Bone | 2 | 5 |
| Others | 9 | 4 |
| No of metastatic sites | | |
| 1 | 5 | 4 |
| 2 | 6 | 6 |
| > 2 | 4 | 7 |

while 13 (76.5%) in the GM-CSF + DTIC arm had a WHO grade 3 or 4 toxicity (Table 2).

Fever (32 patients), decrease in performance status (29 patients) and pain (23 patients) were the most common adverse events related to GM-CSF in the 2 groups. Only 3 patients had a grade 4 toxicity with anaemia (2 pts) and vomiting (1 pt), while 6 patients in the GM-CSF arm and 10 in the GM-CSF + DTIC arm had a grade 3 toxicity. The grade 3 toxicities were fever (6 patients in both groups), pain (6 patients in the GM-CSF + DTIC arm; 1 in the GM-CSF arm), decrease in performance status (7 patients in the GM-CSF + DTIC arm; 3 in the GM-CSF arm), dyspnoea (4 patients in the GM-CSF arm) and thrombocytopenia (2 patients in the GM-CSF + DTIC arm).

### Response to treatment and survival

15 were included in the GM-CSF arm while 17 were included in the GM-CSF + DTIC arm. Among 30 assessable patients, no patient achieved an objective response. The best response achieved was stable disease in 3 patients treated with GM-CSF + DTIC, while all other patients already had tumour progression at the evaluation done after 2 cycles of treatment.

Among 8 patients who received DTIC alone after failure with GM-CSF alone, one patient had a partial response.

The median follow-up was 44.2 months. The median overall survival was 6.3 months; 6.3 months in the GM-CSF arm and 7.3 months in the GM-CSF + DTIC arm.

### DISCUSSION

In this study, GM-CSF induced no clinically evaluable antitumoural activity in patients with metastatic melanoma, given either alone or in association with standard chemotherapy by

**Table 2** Grade 3–4 WHO toxicity

| | GM-CSF | | GM-CSF + DTIC | |
|---|---|---|---|---|
| Toxicity | Gr 3 | Gr 4 | Gr 3 | Gr 4 |
| Fever | 6 | 0 | 6 | 0 |
| Pain | 1 | 0 | 6 | 0 |
| Decrease in performance status | 3 | 0 | 7 | 0 |
| Dyspnea | 0 | 0 | 4 | 0 |
| Skin disorders | 0 | 0 | 0 | 0 |
| Nausea / vomiting | 0 | 0 | 0 | 1 |
| Hypotension | 0 | 0 | 0 | 0 |
| Neurological | 3 | 0 | 0 | 0 |
| Anaemia | 0 | 0 | 1 | 2 |
| Thrombocytopenia | 0 | 0 | 2 | 0 |
| Hypercreatininaemia | 0 | 0 | 0 | 0 |

dacarbazine. This is the first study reporting the clinical evaluation of GM-CSF alone or in association with chemotherapy in metastatic melanoma patients.

Other authors have shown a dramatic increase in response rate with subcutaneous GM-CSF associated with chemotherapy (ifosfamide, cisplatinum and doxorubicin) in patients with sarcoma (Edmonson et al, 1994, 1997). However our study does not confirm that GM-CSF increases the efficacy of a given chemotherapy in metastatic solid tumours.

In the phase I trial reported by Edmonson, 15 patients were treated with chemotherapy supposed to induce 30% objective response and complete response less than 5%. 14 patients had metastatic localization. An objective response was seen in 7 patients. Furthermore, 5 patients had a complete remission and another had a complete remission on metastatic site but not on the primary tumour. The median duration of survival was 27 months, longer than expected. Another phase II study from the EORTC showed in 104 patients with metastatic sarcoma, that GM-CSF added to escalated doses of chemotherapy could increase the expected response rate to 45% and 10% of complete response (Steward et al 1993); nevertheless, the impact of GM-CSF as well as the dose intensity of chemotherapy are points to be debated. Except for sarcomas, where the chemotherapy used is highly effective (objective response ≥ 30%), the wide literature so far on the topic of GM-CSF given to prevent haemotoxicity, has not provided data suggesting tumour efficacy of GM-CSF in metastatic solid tumours.

Our findings do not exclude the possibility of GM-CSF having antitumoural activity on a lower tumour burden or in association with a treatment devoted to triggering an immune response. This hypothesis has since received confirmatory data on the ability of GM-CSF to enhance immune response, even though there is not yet a major impact on clinical outcome. The main focus of GM-CSF activity on immune response has concerned the impact of GM-CSF on the recruitment, activation and survival of dendritic cells. It is known that GM-CSF given by intradermal route, leads to local skin reactions, with a progressive accumulation of Langerhans cells, while the subcutaneous route failed to do so (Kaplan et al, 1992). It has been shown that GM-CSF enhances the recruitment of dendritic cells, thereby increasing the number of dendritic cells in regional lymph nodes, providing contact with lymphocytes (Kass et al, 2001). In addition, GM-CSF enhances the activation and survival of dendritic cells (Armitage, 1998). Moreover, GM-CSF protects dendritic cells from apoptotic death (Rabinovich et al, 1999).

*© 2001 Cancer Research Campaign* 

The induction of an immune response by GM-CSF given intradermally can be detected in eliciting local skin reaction and strong DTH response, when used alone (Kaplan et al, 1992) or as adjuvant therapy with peptide (Jäger et al, 1996; McNeel et al, 1999), a finding already demonstrated in experimental models (Disis et al, 1996).

Furthermore, there are arguments for stimulation of T lymphocytes. When the GM-CSF was given intralesionally in subcutaneous metastases, an increase in T cells, especially CD4+ cells was noticed at the tumour site (Miller et al, 1981). Added to vaccination procedures, either to peptide (Jäger et al, 1996) or to irradiated autologous tumour cells (Si et al, 1996), GM-CSF induced an increase in the CTL response.

The increase in immune response, as shown biologically, has been associated in clinical trials with tumour response.

It has been shown that GM-CSF given intralesionally in subcutaneous metastases induced 3 partial responses in 13 patients with metastatic melanoma (Miller et al, 1981). GM-CSF associated with vaccination either with peptide or with irradiated autologous tumour cells has shown to increase the clinical response to peptide alone (Jager et al, 1996) or to irradiated autologous tumour cells with BCG (Si et al, 1996). GM-CSF given intradermally with peptides derived from Melan A/MART-1, tyrosinase and gp 100 in HLA-A2+ patients with metastatic melanoma induced one complete response and 2 partial responses in 3 patients, while vaccination with the same peptide alone induced only stabilization. Furthermore, in 20 patients with metastatic melanoma treated by vaccination with autologous irradiated tumour cells associated with BCG and GM-CSF, 2 patients had a complete response and 2 others a partial response, while a prior study by vaccination with autologous irradiated tumour cells associated with BCG did not show any clinical response (Leong et al, 1999). In the latter study (Leong et al, 1999), GM-CSF added to a treatment designed to trigger an immune response, was effective for large metastatic tumour burden from melanoma.

On the other hand, another study demonstrated for efficacy with GM-CSF in a lower tumour burden, while given as adjuvant to surgery stage III or IV melanoma patients, but the results have to be taken with caution (Spitler et al, 2000). 48 patients with stage III or IV melanoma rendered disease-free by surgery received GM-CSF in an adjuvant setting. Compared with matched historical controls, overall survival and disease-free survival were significantly prolonged in patients receiving GM-CSF. The major concern of the Spitler study is that it is an uncontrolled trial, even compared to matched historical controls. The metastatic patients in it were from a very selected population, rendered macroscopically disease-free by surgery, which is far from the general population, especially patients included in our study. No conclusion can be drawn out from the study, except that immunobiologic modifier drugs are probably more active in low tumour burden.

The toxicity induced by the dosage and schedule in this study was similar to that reported in the initial sarcoma study with identical dosage and schedule (Edmonson et al, 1994, 1997). In both arms of our study, fever, decrease in performance status and pain were the most frequent grade 3 side effects for patients accounting for 38.7%, 32.3% and 19.4% respectively. In the initial report (Edmonson et al, 1994), toxicities were reported without grading of severity. Nevertheless, 10 of the 15 patients had to receive prednisone to allow continuation of treatment.

### CONCLUSION

In this study of patients treated for metastatic melanoma, GM-CSF alone or with dacarbazine, at the dosage and schedule used, did not induce any objective response. Toxicity was severe with 19 patients (59.3%) presenting at least a WHO grade 3 toxicity. Therefore, these findings do not support the contention that GM-CSF has an antitumoural activity in metastatic solid tumours, at least where there is a large tumour burden.

### ACKNOWLEDGMENTS

The authors are indebted to the nurses of the Department of Medical Oncology of Institut Bergonié, Institut Claudius Regaud and Hôpital Saint-André who provided excellent and compassionate care, and Christine Dupouy, Marie-Christine Durand for analysis of this study, and Dorothée Quincy for secretarial assistance. GM-CSF was provided by Novartis, France.

### REFERENCES

Armitage JO (1998) Emerging applications of recombinant human granulocyte-macrophage colony-stimulating factor. *Blood* **92**: 4491–4508

Atkins MB, Lotze MT, Dutcher JP, Fisher RI, Weiss G, Margolin K, Abrams J, Sznol M, Parkinson D, Hawkins M, Paradise C, Kunkel L and Rosenberg SA (1999) High-dose recombinant interleukin 2 therapy for patients with metastatic melanoma: analysis of 270 patients treated between 1985 and 1993. *J Clin Oncol* **17**: 2105–2116

Balch CM, Reintgen DS, Kirkwood JM, Houghton A, Peters L and Kian Ang K (1997) Cutaneous melanoma. In: *Cancer principles and practice in oncology* 5th edition De Vita VT, Hellman S, Rosenberg SA (eds) pp 1947–1994. Philadelphia: Lippincott

Bender A, Sapp M, Schuler G, Steinman RM and Bhardwaj N (1996) Improved methods for the generation of dendritic cells from non proliferating progenitors in human blood. *Immunol Methods* **196**: 121–131

Coulon V, Ravaud A, Gaston R, Delaunay M, Pariente JL, Verdier D, Scrivante V and Gualde N (2000) *In vitro* immunization of patient T cells with autologous bone marrow antigen presenting cells pulsed with tumour lysates. *Int J Cancer* **88**: 783–790

Demirer T, Buckner CD and Bensinger WI (1996) Optimization of peripheral blood stem cell mobilization. *Stem Cells* **14**: 106–116

Disis ML, Bernhard H, Shiota FM, Hand SL, Gralow JR, Huseby ES, Gillis S and Cheever MA (1996) Granulocyte-macrophage colony-stimulating factor: an effective adjuvant for protein and peptide-based vaccines. *Blood* **88**: 202–210

Dranoff G, Jaffee E, Lazenby A, Golumbek P, Levitsky H, Brose K, Jackson V, Hamada H, Pardoll D and Mulligan RC (1993) Vaccination with irradiated tumor cells engineered to secrete murine GM-CSF stimulates potent, specific and long lasting anti-tumor immunity. *Proc Natl Acad Sci USA* **90**: 3539–3543

Edmonson JH, Long HJ and Kvols LK (1994) Cytotoxic drugs plus subcutaneous granulocyte-macrophage colony-stimulating factor: can molgrastim enhance antisarcoma therapy? *J Natl Cancer Inst* **86**: 312–314

Edmonson JH, Long HJ, Kvols LK, Mann BS and Grill JP (1997) Can molgrastim enhance the antitumour effect of cytotoxic drugs in patients with advanced sarcoma? *Ann Oncol* **8**: 637–641

Gehan EA (1961) The determination of the number of patients required in a preliminary and a follow-up trial of a new chemotherapeut agent. *J Chron Dis* **13**: 346–353

Grabstein KH, Urdal DL, Tushinski RJ, Mochizuki DY, Price VL, Cantrell MA, Gillis S and Conlon PJ (1986) Induction of macrophage tumoricidal activity by granulocyte-macrophage colony stimulating factor. *Science* **232**: 506–508

Greenberg P, Advani R, Keating A, Gulati SC, Nimer S, Champlin R, Karanes C, Gorin NC, Powles RL, Smith A, Lamborn K and Cuffie C (1996) GM-CSF accelerates neutrophil recovery after autologous hematopoietic stem cell transplantation. *Bone Marrow Transplant* **18**: 1057–1064

Hill AD, Redmond HP, Austin OM, Grace PA and Bouchier-Hayes D (1993) Granulocyte-macrophage colony-stimulating factor inhibits tumor growth. *Br J Surg* **80**: 1543–1546

Ho RC (1995) Medical management of stage IV malignant melanoma. *Cancer* **75 (Suppl 2)**: 735–741

 *© 2001 Cancer Research Campaign*

Jäger E, Ringhoffer M, Dienes HP, Arand M, Karbach J, Jager D, Ilsemann C, Hagedorn M, Oesch F and Knuth A (1996) Granulocyte-macrophage-colony-stimulating factor enhances immune responses to melanoma-associated peptides *in vivo. Int J Cancer* **67**: 54–62

Kaplan G, Walsh G, Guido LS, Meyn P, Burkhardt RA, Abalos RM, Barker J, Frindt PA, Fajardo TT, Celona R et al (1992) Novel response of human skin to intradermal recombinant granulocyte/macrophage-colony-stimulating factor: Langerhans cell recruitment, keratinocyte growth, and enhanced wound healing. *J Exp Med* **175**: 1717–1728

Kass E, Panicali DL, Mazzara G, Schlom J and Greiner JW (2001) Granulocyte/macrophage-colony stimulating factor produced by recombinant avian poxviruses enriches the regional lymph nodes with antigen-presenting cells and acts as an immunoadjuvant. *Cancer Res* **61**: 206–214

Kirkwood JM, Strawderman MH, Ernstoff MS, Smith TJ, Borden EC and Blum RH (1996) Interferon alfa-2b adjuvant therapy of high-risk resected cutaneous melanoma: the Eastern Cooperative Oncology Group trial EST 1684. *J Clin Oncol* **14**: 7–17

Kushner BH and Cheung NKV (1989) GM-CSF enhances 3F8 monoclonal antibody-dependent cellular toxicity against human melanoma and neuroblastoma. *Blood* **73**: 1936–1941

Legha SS, Ring S, Eton O, Bedikian A, Buzaid AC, Plager C and Papadopoulos N (1998) Development of a biochemotherapy regimen with concurrent administration of cisplatin, vinblastine, dacarbazine, interferon alfa, and interleukin-2 for patients with metastatic melanoma. *J Clin Oncol* **16**: 1752–1759

Leong SP, Enders-Zohr P, Zhou YM, Stuntebeck S, Habib FA, Allen RE, Sagebiel RW, Glassberg AB, Lowenberg DW and Hayes FA (1999) Recombinant human granulocyte macrophage colony stimulating factor (rh GM-CSF) and autologous melanoma vaccine mediate tumor regression in patients with metastatic melanoma. *J Immunother* **22**: 166–174

Mazumder A, Agah R and Charak B (1990) Novel use of GMCSF as an antitumour immune potentiator after transplantation. *Blood* **76**(**Suppl 1**): abstr 2200

McNeel DG, Schiffman K and Disis ML (1999) Immunization with recombinant human granulocyte-macrophage colony-stimulating factor as a vaccine adjuvant elicits both a cellular and humoral response to recombinant human granulocyte-macrophage colony-stimulating factor. *Blood* **93**: 2653–2659

Metcalf D (1985) The granulocyte-macrophage stimulating factors. *Science* **229**: 16–22

Miller AB, Hoogstraten B, Staquet M and Winkler A (1981) Reporting results of cancer treatment. *Cancer* **47**: 207–214

Nagler A, Shur L, Or R, Barak V, Condiotti R and Fabian I (1994) Granulocyte-macrophage colony-stimulating factor (GM-CSF) dependant monocyte-mediated cytotoxicity post-autologous bone marrow transplantation. *Exp Hematol* **22**: 780 (abst 385)

Rabinovich GA, Riera CM and Iribarren P (1999) Granulocyte-macrophage colony-stimulating factor protects dendritic cells from liposome-encapsulated dichloromethylene diphosphonate-induced apoptosis through a Bcl-2-mediated pathway. *Eur J Immunol* **29**: 563–570

Ragnhammar P, Fagerberg J, Frodin JE, Hjelm AL, Lindemalm C, Magnusson I, Masucci G and Mellstedt H (1993) Effect of monoclonal antibody 17-1A and GM-CSF in patients with advanced colorectal carcinoma. Long lasting complete remissions can be induced. *Int J Cancer* **53**: 751–758

Ravaud A, Chevreau C, Cany L, Houyau P, Dohollou N, Roche H, Soubeyran P, Bonichon F, Mihura J, Eghbali H, Tabah I and Bui BN (1998) Granulocyte-macrophage colony-stimulating factor in patients with neutropenic fever is potent after low-risk but not after high-risk neutropenic chemotherapy regimens: results of a randomized phase III trial. *J Clin Oncol* **16**: 2930–2936

Richard C, Alsar MJ, Calavia J, Bello-Fernandez C, Baro J, Loyola I, Rios R, Cuadrado MA, Gonzalez-Pardo C, Iriondo A, Conde E and Zubizzarreta A (1993) Recombinant human GM-CSF enhances T cell mediated cytotoxic function after ABMT for hematological malignancies. *Bone Marrow Transplant* **11**: 473–478

Santoli D, Clark SC, Kreider BL, Maslin PA and Rovera G (1988) Amplification of IL-2 driven T cell proliferation by recombinant human IL-3 and granulocyte-macrophage colony-stimulating factor. *J Immunol* **141**: 519–526

Si Z, Hersey T and Coates AS (1996) Clinical responses and lymphoid infiltrates in metastatic melanoma following treatment with intralesional GM-CSF. *Melanoma Res* **6**: 247–255

Sieff CA (1987) Hematopoietic growth factors. *J Clin Invest* **79**: 1549–1557

Spitler LE, Grossbard ML, Ernstoff MS, Silver G, Jacobs M, Hayes FA and Soong SJ (2000) Adjuvant therapy of stage III and IV malignant melanoma using granulocyte-macrophage colony-stimulating factor. *J Clin Oncol* **18**: 1614–1621

Steward WP, Verweij J, Somers R, Spooner D, Kerbrat P, Clavel M, Crowther D, Rouëssé J, Tursz T, Tueni E et al (1993) Granulocyte-macrophage colony-stimulating factor allows safe escalation of dose-intensity of chemotherapy in metastatic adult soft tissue sarcomas: a study of the European Organization for Research and Treatment of Cancer Soft Tissue and Bone Sarcoma Group. *J Clin Oncol* **11**: 15–21

Szabolcs P, Moore MA and Young JW (1995) Expansion of immunostimulatory dendritic cells among myeloid progeny of human CD34+ bone marrow precursors cultured with c-kit ligand, granulocyte-macrophage colony-stimulating factor, and TNF-alpha. *J Immunol* **154**: 5851–5861

Update of recommendations for the use of hematopoietic colony-stimulating factors: evidence-based clinical practice guidelines (1996) American Society of Clinical Oncology. *J Clin Oncol* **14**: 1957–1960

Williams MA, Kelsey SM, Collins PW, Gutteridge CN, Bernard T and Newland AC (1994) *Ex vivo* augmentation of monocyte activation and antitumor potential of GM-CSF (Molgrastim) following intensive chemotherapy. *Br J Hematol* **86**: **(Suppl 1):** 20 (abstr 72)

Willman CL, Stewart CC, Miller V, Yi TL and Tomasi TB (1989) Regulation of MHC class II gene expression in macrophages by hematopoietic colony-stimulating factors (CSF). Induction by granulocyte/macrophage CSF and inhibition by CSF-1. *J Exp Med* **170**: 1559–1567

*© 2001 Cancer Research Campaign*
